# A quarter of a century of job transitions in Germany^☆^

**DOI:** 10.1016/j.jvb.2013.11.001

**Published:** 2014-02

**Authors:** Ralph Kattenbach, Thomas M. Schneidhofer, Janine Lücke, Markus Latzke, Bernadette Loacker, Florian Schramm, Wolfgang Mayrhofer

**Affiliations:** a Tongji University, Sino-German School of Postgraduate Studies, Shanghai, China; b WU Vienna University of Economics and Business, Austria; c University of Hamburg, Germany; d Lund University, School of Economics and Management, Sweden

**Keywords:** Career, Job transition, GDP, Age, Qualified employees

## Abstract

By examining trends in intra-organizational and inter-organizational job transition probabilities among professional and managerial employees in Germany, we test the applicability of mainstream career theory to a specific context and challenge its implied change assumption. Drawing on data from the German Socio-Economic Panel (GSOEP), we apply linear probability models to show the influence of time, economic cycle and age on the probability of job transitions between 1984 and 2010. Results indicate a slight negative trend in the frequency of job transitions during the analyzed time span, owing to a pronounced decrease in intra-organizational transitions, which is only partly offset by a comparatively weaker positive trend towards increased inter-organizational transitions. The latter is strongly influenced by fluctuations in the economic cycle. Finally, the probability of job transitions keeps declining steadily through the course of one's working life. In contrast to inter-organizational transitions, however, this age effect for intra-organizational transitions has decreased over time.

## Highlights

• We analyze developments in job transitions in Germany from 1984 to 2010.
• Against the common belief of increased mobility, there is a negative trend in the level of job transitions.
• The negative trend is due to a pronounced decrease in internal and a weak increase in external transitions.
• External job transitions are more influenced by the GDP than by a global trend over time.
• The probability of job transitions declines with age. For internal transitions, this age effect has declined over time.

## 1 Introduction

Since the mid-1980s, Western societies have been witnessing changes in the organization of work, careers, and employment relations. These changes are often related to a paradigm shift from “Fordistic” to “post-Fordistic” forms of organization (Opitz, 2004) or a shift from “bureaucratic” to “post-bureaucratic” or “entrepreneurial” modes of work and career regulation (du Gay, Salaman, & Rees, 1996). The effects of socio-cultural and politico-economic transformation processes have been differently evaluated in work, employment and organization studies (Courpasson and Reed, 2004, McCabe, 2009, Roper et al., 2010), in sociology (Bauman, 2000, Boltanski and Chiapello, 2006) and in contemporary management and career studies (Arthur and Rousseau, 1996b, Kanter, 1997).

Concerning the latter, metaphors like the boundaryless (Arthur, 1994), protean (Hall, 1996), post-corporate (Peiperl & Baruch, 1997), chronically flexible (Iellatchitch, Mayrhofer, & Meyer, 2003), or kaleidoscope career (Maniero & Sullivan, 2005) aim to reflect these developments. In a nutshell, they tend to emphasize increased individual freedom and autonomies in individuals' careers, which now allegedly develop beyond the constraints of single organizations (Inkson, 2006, p. 49) which provided “traditional” careers. Due to technological changes, organizational restructuring, layoffs, and demographical developments, individuals are increasingly pushed and pulled into a changing career world. Numerous authors expect radical changes in employment patterns due to these developments (Giesecke & Heisig, 2010, p. 406).

Recently, scholars have issued a call to “tackle the next stage of the research cycle, i.e. empirical study” (Dries & Verbruggen, 2012, p. 269). One quantifiable indicator for changing career environments is the number of job transitions, which is said to be soaring (Rodrigues & Guest, 2010). But there are substantial doubts concerning the extent of job mobility (Chudzikowski, 2012, Guest and McKenzie-Davey, 1996) and some literature even claims that no fundamental change in career behavior and career perceptions has occurred at all (e. g., Diewald and Sill, 2005, Jacoby, 1999, Kattenbach et al., 2011). In addition, while the literature on new careers tends to acknowledge contextual and institutional factors, less importance is given to these factors empirically (Mayrhofer, Steyrer, & Meyer, 2007). This raises two questions. First, do these concepts, which have mainly been developed in the Anglo-American context, adequately describe what is happening in different institutional environments such as Germany, which is arguably different in terms of dismissal protection, social insurance institutions, education system, and industrial relations (Hall & Soskice, 2001)? Second, to what extent is the underlying change assumption in career studies (Mayrhofer, 2012) a valid one?

The paper at hand addresses these questions and analyzes trends of transition probabilities among qualified employees in West Germany between 1984 and 2010. We concentrate on this group, defined as employees with managerial tasks and or other highly qualified jobs, because it provides a good test bed for the change hypothesis. Highly qualified employees are more difficult to replace, and they enjoy better job conditions (e.g. in terms of autonomy, power and wage level). Within the context outlined above they should perceive fewer economic boundaries and more opportunities for job changes (Powell and Maniero, 1999, Sherer et al., 1987). We focus on Germany to consider a context with rather rigid institutional structures, characterized by stable welfare institutions and more powerful unions than those in the US (Biemann, Fasang, & Grunow, 2011). We will rely on data from West Germany only, for the assumed change in the career context beginning in the 1980s, and data from East Germany are only available from 1991 on.

Our paper contributes to the career literature in three ways: First, by using representative panel data and longitudinal analyses, it seeks to contribute to the reinvigoration of the study of careers (Savickas, 2002). Second, it challenges the change and universality assumption prevalent in much of career research (Chudzikowski, 2012, Collin, 1998, Mayrhofer, 2012). Although we acknowledge that change takes place, we argue that the change assumption prevalent in careers research may be exaggerated, and that it cannot be generalized across different national contexts. Hence, we do not contest the conceptual quality of mainstream careers theories, most of all the boundaryless career theory, but the universality of their application. Third, and linked with this, the paper points out the relevance of contextual influences exemplified in a highly regulated economy, and it endorses the recently advocated discussion on the impact of institutional factors (Biemann et al., 2012, Inkson et al., 2012).

## 2 Theoretical background

Two conceptualizations of careers, which reflect the above-mentioned developments and put particular emphasis on the agentic quality of career trajectories, have gained substantial attention in career research and represent the contemporary mainstream: protean careers (Hall, 1996, Hall and Mirvis, 1996), on the one hand, and boundaryless careers (Arthur & Rousseau, 1996b; for a comparison see Briscoe, Hall, & DeMuth, 2006) on the other. The former is characterized by careers developing independently of traditional career arrangements (Segers, Inceoglu, Vloeberghs, Bartram, & Henderickx, 2008). It relies on a conception of psychological success resulting from individual career management, as opposed to career planning and development arranged by the organization. Protean careers have been characterized as involving greater mobility, a more holistic life perspective, and a developmental progression (Hall, 1996).

The boundaryless career concept is framed in a similar way. Introduced in the mid-1990s and since then increasingly discussed (see e.g. Arthur et al., 2005, Sullivan and Arthur, 2006) and refined (Arthur, 2008, Tams and Arthur, 2010), the boundaryless career is conceptualized as emancipation from organizations, which used to provide traditional ways of development implying a logic of vertical coordination and long-term commitment. It is therefore described as “the opposite of the ‘bounded’, or organizational career” (Arthur & Rousseau, 1996a, p. 3) and characterized by increased boundary crossing by the career actor (Sullivan, 1999), who may perceive a boundaryless future regardless of structural constraints (Arthur & Rousseau, 1996a, p. 5). According to the latter, the boundaryless career implies not only a mobility in terms of job transitions but also a psychological mobility (Sullivan and Arthur, 2006, Verbruggen, 2012). However, in the following we focus on physical mobility.

Both the protean career and the boundaryless career concept share, among other things, the idea of physical mobility across jobs, functions and organizations (Briscoe et al., 2006, Sullivan and Baruch, 2009). Hence, when these career concepts increase in importance, one may presume both an overall increase in inter-organizational job transitions, meaning job-to-job changes between organizations (for the concept of work role transitions see, e.g. Nicholson, 1984), and an overall decrease in internal job transitions, broadly defined as any essential change in task and duty within an organization, in particular across hierarchical, functional, and inclusion boundaries (Schein, 1971). For the remainder of this study, we will employ the term “external” to denote inter-organizational job transitions, and “internal” to denote intra-organizational job transitions.

## 3 Hypotheses

Despite theoretical claims about the overall increase of job transitions due to boundaryless and protean careers, admittedly the empirical support for such a rise is modest at best (Chudzikowski, 2012, Pringle and Mallon, 2003). Job tenure and turnover have remained stable in several parts of the world (Rodrigues & Guest, 2010: 1168) and with each successive generation (Lyons, Schweitzer, Ng, & Kuron, 2012). Recently, Rodrigues and Guest (2010) analyzed data from the OECD Employment Statistics Database in order to capture historical trends in job stability for the years 1992 to 2006. They find few changes for job tenure and turnover in the US, Japan and Europe, and there is no increase in job transitions among qualified employees. Their results for Germany indicate a moderate increase in job tenure for the German labor market between 2000 and 2006. This corresponds to a decreased mobility rate between 1974 and 1994 drawn from an analysis of the GSOEP (Winkelmann & Zimmermann, 1998). A more detailed analysis reveals some variations as job tenure declined moderately among men between 15 and 24, low-tenured workers and men between 55 and 64. In addition changes for women older than 25 could be observed, as their tenure increased (Rodrigues & Guest, 2010). Likewise, empirical studies show that the number of job transitions (Diewald & Sill, 2005) and tenure (Auer & Cazes, 2000) was fairly constant in the 1990s. Therefore one should not expect a general linear trend reflecting an increase in boundary crossing probabilities. This particularly holds true for countries with rather rigid structural boundaries like Germany (Biemann et al., 2011). Data shows that the willingness to change jobs in Germany has been rather constant over time (Kattenbach et al., 2011).

Gunz, Evans, and Jalland (2000, p. 24) conclude that “the more that boundaries are knocked down, it seems, the more people put up new ones”. Consequently, we do not expect a general trend towards more transitions. Rather, we postulate certain inertia in their overall level in Germany.

Hypothesis 1 Job transition probability among professional and managerial employees in West-Germany remains on a stable level between 1984 and 2011.

Empirical evidence shows that the business cycle explains much variance in career-related behavior such as job mobility (Cornelißen et al., 2007, Diebold et al., 1996, Diebold et al., 1997). Economic factors influence the expansion or downsizing of companies (Ng, Sorensen, Eby, & Feldman, 2007) and career patterns (Biemann et al., 2012). A longitudinal study conducted in Australia revealed that job security was the only organizational factor related to career change (Carless & Arnup, 2011). Hence we postulate that oscillations in the level of transitions are influenced by the macroeconomic climate, as measured by GDP.

Hypothesis 2 Variations in the business cycle influence yearly oscillations in the level of job transitions among professional and managerial employees in West-Germany between 1984 and 2011.

Career stage and age are a well-known factor when looking at various career outcomes. The career literature offers many age-related conceptualizations of careers with varying numbers of stages and cut-off points but very similar patterns for career developments (e.g. Gould, 1978, Levinson, 1978, Levinson, 1996, Munley, 1977, Vaillant, 2002). Different levels of job transitions are reported for the single career-stages, e.g. with a peak in the starting and mid-career phase and a decrease with further career progress (e.g. Lyons et al., 2012, p.349). The frequency of external job mobility has a significant positive effect on salary earned in the US and in Hong Kong (Lam, Ng, & Feldman, 2012), although this effect is moderated by the individual's career stage: The positive effects are stronger for early-career workers than for mid- and late-career workers. More general and detached from individual career progress, age has been recognized as a fundamental explanatory factor for turnover decisions (e.g. Mowday et al., 1982, Sullivan and Crocitto, 2008). Empirical work on job transitions shows that younger managers are more mobile than older managers (Biemann et al., 2012, Groot and Verberne, 1997, Ng and Feldman, 2009, Nicholson and West, 1988, O'Brien, 2007). In accordance with this repeatedly confirmed finding, we hypothesize that the level of job transitions decreases with age.

Hypothesis 3a The probability of job transitions among professional and managerial employees in West-Germany decreases with age between 1984 and 2011.

There are contextual developments with the potential of challenging this relationship between age and job transitions, however. First, with the current increase in the mean and average ages of people in developed countries, they tend to stay in the workforce longer (Feldman, 2007). This implies potentially more transitions in later career stages as well. Second, as the organizational career jungle-gym (Gunz, 1988) is becoming increasingly flatter, longer job duration for late career stages bears potential consequences for early career stages as well, decreasing available space. Hence, the literature might suggest that the age effect outlined in Hypothesis 3a declines over time.

In Germany, however, this appears unlikely. As far as the first objection is concerned, German labor law managing entitlement to retire is rather rigid, and has changed only recently in 2012, allowing for more flexibility. It offers relatively high security for employees, such as enhanced dismissal protection with progressing seniority and – at least up to now – a comparably well-equipped public and occupational pension scheme. Second, the early retirement rate has been fairly constant over the years (OECD, 2003, OECD, 2011). The Germans – like many populations in Western Europe such as the Austrian, Italian, or French – seem to regard retirement as their sacred right which they intend to realize as soon as possible (Vance & Paik, 2011). Third, the compensation plans based on seniority make people in later career stages more expensive for organizations. This leads to special incentive schemes for realizing early retirement as well (Lücke, Kattenbach, & Schramm, 2013).

As far as the second objection is concerned, one has to remember that the baby boomer generation (defined as the post-World War II cohort born between 1946 and 1964) responsible for this potential problem (Collin, 2003), has begun to retire in larger numbers only at the end of the first decade of the millennium. Hence, problems of replacement in the labor market will only be likely from then on, and related effects have not affected our sample.

As a result, we postulate that for Germany the decreasing probability of job transition with increasing age does not change significantly over time.

Hypothesis 3b The age effect among professional and managerial employees in West-Germany remains constant between 1984 and 2010.

## 4 Sample, variables and methods

Our study uses data of the German Socio-Economic Panel (GSOEP), 2012 release (Wagner, Frick, & Schupp, 2007). The analysis focuses on West Germany to ensure continuity of the societal framework across the whole period from 1984 to 2010. The GSOEP is a longitudinal household-based panel survey which has been carried out every year since 1984. As a representative set of individual data in Germany, the GSOEP provides a platform for examining not only objective socio-demographic and economic measures but also information concerning personality and the subjective perceptions of working conditions. The GSOEP data have been widely used to investigate the individual effects of job instability (e.g. Bethge et al., 2008, Boerner and Schramm, 1998, Clark et al., 2008, Lurweg, 2010, Schramm, 1992). Other studies with various foci also took advantage of the GSOEP. They include downward mobility (Pollmann-Schult, 2006), the effect of economic growth and the unemployment rate on job transitions (Cornelißen et al., 2007) or changes in career patterns (Biemann et al., 2012). Even changes in internal (Diewald & Sill, 2005) and external job transitions have been investigated (Giesecke & Heisig, 2010), but none of the mentioned studies has focused either on the boundaryless career concept or on the specific group of qualified employees to which the predictions of the boundaryless career should especially apply. Furthermore with an observation period which grows year by year, the GSOEP dataset has become more appropriate to analyze long-term trends as implied by the emergence of boundaryless careers. The following variables have been considered to test our hypotheses:

*Job transitions.* Detailed information on job transitions is available from survey year 1985 on. Participants in the GSOEP are asked on a yearly basis whether they have changed their job position during the last year. Since this information refers to the previous year, we have predated the information on transitions to the referred year. The initial question is followed by several items on the nature of and reason for the job change. We have derived two dichotomous variables indicating internal job transitions (“I have changed positions within the same company”) and external ones (“I have started a new position with a different employer”).

*Business cycle.* To indicate the business cycle, we have used the regional gross domestic product growth rate in real terms for West Germany. The data from 1983 to 1991 is drawn from the Federal Ministry of Labor and Social Affairs (BMAS); from 1992 to 2010, we used the data from the Federal Statistical Office and statistical offices of the states (*Statistische Ämter des Bundes und der Länder*). As Cornelißen et al. (2007) have shown, the pattern of seasonal changes in the GDP has been repeated over the years. Thus, for considering a trend over several years, seasonal changes can be disregarded, or more precisely, subsumed under their respective years. It is reasonable that the effect of economic developments on the labor market has a certain delay. To cover the direct as well as the lagged economic effect, we have included a GDP variable for the year of job transition and a lagged GDP variable indicating the economic growth in the year before the transition took place.

*Age and sex category.* In postulating a major effect of age on the occurrence of job transitions, we have generated an age variable referring to the reported year (previous to the survey year). Age has also been used as a demarcation variable for the sample of qualified employees. Only employees aged 20 to 63 in the reported year have been included in our analyses. The lower end is to exclude people without sufficient vocational experience and/or education to reasonably belong to the group in question. The upper end has been chosen due to the retirement age in real terms in Germany. Although the legal retirement age is 65 for both sexes, the effective average age for retirement is 61.8 for men, and 60.5 for women (Statista, 2013).

For multi-level analyses the year dates and the age variable have been centralized to the lower bounds of 1984 and 20 years respectively. Regression coefficients can therefore be interpreted as an increase in the percentage probability with each additional year in the course of time and in one's working life respectively. GDP regression coefficients can be interpreted as an increase in the percentage probability with each percentage point of economic growth.

Sex category is operationalized as a dichotomous variable with 0 ‘male’ and 1 ‘female’.

*Sample.* The sample includes 5688 qualified employees. Respondents who have reached a managerial or professional position once remain in this status group on the average for a period of $\bar{M}=4.8\;\mathrm{years}$ (*SD* = 4.85). This results in N = 24 956 observations. The size of the group of respondents for each year ranges from a minimum N_1987_ = 396 up to N_2002_ = 2219. Table 1 provides an overview on sample size, gender and job transition rates summarized for 5-year periods (with the final period, however, being six years in duration). The second column N_acc_ indicates the accumulated number of responses for each period whereas Ø N_wght_/year estimates the average yearly population size for each period. Sample descriptives are weighted by a grossing-up factor to extrapolate results from the GSOEP sample and project them onto the population of all qualified employees in West Germany.

**Table 1:** Descriptive statistics for professional and managerial employees in West Germany, weighted.

Report years^a^	N_acc_	Ø N_wght_ per year	Ø age	Prop. of women	Job trans.	Int. trans.	Ext. trans.	Males			Females		
								Job trans.	Int. trans.	Ext. trans.	Job trans.	Int. trans.	Ext. trans.
1984–2010	24 956	15 689 863	41.40	.262	12.61	4.11	8.50	11.61	3.77	7.85	15.51	5.07	10.43
1984–1989	2550	2 354 911	41.18	.207	13.84	6.02	7.87	12.68	5.36	7.38	18.29	8.55	9.75
1990–1994	2708	3 424 068	40.50	.219	13.68	5.29	8.39	12.57	4.95	7.62	17.62	6.49	11.13
1995–1999	4063	3 991 963	41.29	.230	13.48	3.82	9.66	12.47	3.88	8.59	16.87	3.62	13.25
2000–2004	7256	3 866 545	41.57	.266	10.98	3.10	7.88	10.13	2.49	7.63	13.34	4.77	8.57
2005–2010	8379	4 407 287	41.78	.320	12.57	3.88	8.69	11.39	3.52	7.87	15.08	4.64	10.44

Data of pooled cross-sectional analysis. All job transition rates are given in %. *acc*: accumulated; *wght*: weighted.

a Survey years range from 1985–2011 providing information on the job situation in the preceding years (1984–2010).

When exploring structural changes in career paths due to a demographic change, the data reveal only little impact on the group of qualified employees. Within two decades the average age has increased by 0.6 years from 41.18 to 41.78. By contrast the rise of a female workforce is much more conspicuous. The proportion of qualified female employees in the predominantly male group has risen by 50% from about one fifth in the late 80s to one third in recent years. Compared to the much discussed but still weak demographic change, this gender-related change in workforce composition is clearly more striking.

*Analysis procedure.* To examine the development of job transitions over time and under the impact of the economic cycle (GDP), age and gender, we have analyzed the panel data surveyed from 1985 to 2011. For dichotomous dependent variables like the occurrence of a job transition, the multilevel *linear probability model* (LPM) is an appropriate analytical tool, although there are two shortcomings. First, predicted probabilities are not restricted to the range of 0 to 1; higher and negative outcomes are possible. Due to limited variance of the independent measures (e.g. GDP and sex) this limitation does not strongly affect our analyses. The second often mentioned shortcoming is that marginal probabilities are constant in LPM and therefore do not depend on the values of the explanatory variables. However, for the independent variables used in our models, we precisely assume constant marginal probabilities, e.g. with age, GDP or the course of time. Additionally, the coefficients of a probit model would be much more difficult to interpret for continuous regressors (Wooldridge, 2009: 250).

For job transitions in general, and for internal and external transitions in particular, we have carried out multilevel linear probability models to predict the impact of the independent variables on the probability that a job transition takes place (Wooldridge, 2009). The individual characteristics, age and sex, are considered as respondent-related level 2 variables whereas the course of time and the economic cycle (GDP) on a yearly basis represent macro-characteristics of the economic and societal situation (level 1). Although age is perfectly correlated with the course of time on an individual level, it shows – like the other regressors – no multicollinearity (see VIF values in Table 2), since individuals start survey participation at different points in time and at different ages. Testing structural changes of the age-effect over time, as well as controlling for a changed impact of sex, we have included cross-level interaction terms (year ∗ age and year ∗ sex) in the models.

**Table 2:** Correlation matrix.

		1	2	3	4	5	6	7
1	Transition year	1.346						
2	GDP	−.289	1.101					
3	Lagged GDP	−.409	.039	1.211				
4	Sex	.067	*−.013*	−.026	1.019			
5	Age	.134	−.040	−.059	−.110	1.034		
6	Job transition	−.043	.031	.036	.043	−.207	n.a.	
7	Internal transition	−.051	*.020*	.028	.027	−.098	.536	n.a.
8	External transition	*−.015*	.023	.024	.032	−.177	.812	−.057

N = 24 956; Correlations that are not significant on a 99.9% level (two-tailed) are marked in italic; VIF—values for predictor variables on diagonal.

To find the most appropriate model to fit the data we have compared five different models using likelihood ratio (LR) tests. The five models are a multilevel LPM null model without independent variables, a simple OLS regression null model as an alternative analysis model and three nested linear probability models (LPMs) successively including level 1 and level 2 variables as well as interaction terms of age and sex with time. A significant LR test indicates that the nested models are more appropriate than the less detailed null models.

LR tests reveal that a multilevel probability model using the panel structure of the GSOEP is more appropriate to explain variance in the probability of global job transitions, internal transitions and external transitions than simple OLS regression (*p* < .001 for each of the three dependent variables) and the inclusion of both level 1 and level 2 variables outperform the more restricted models. Table 3 summarizes the LR tests indicating that the LPM models including variables from both levels and the cross-level interaction term are superior to their nested models in explaining general job transitions and internal transitions. Only for external transitions the inclusion of cross-level interaction does not improve the model fit indicating that the interaction between time and age can be ignored due to insignificance. The best fitted models have been chosen to test Hypothesis 1, Hypothesis 2, Hypothesis 3a, Hypothesis 3b for job transitions in general as well as for internal and external transitions.

**Table 3:** Likelihood ratio tests of alternative multilevel linear probability models.

Model fitted	JJ-Trans	Int-Trans	Ext-Trans	Degr. of freedom
	LR test (− 2ΔLL)	LR test (− 2ΔLL)	LR test (− 2ΔLL)	
1. Baseline	na	na	na	na
2. Level 1 var	124.8	76.5	55.5	3
3. Levels 1 & 2 var.	747.0	176.7	531.0	2
4. Levels 1, 2 & cross-level	6.0	8.0	0.9 (n.s.)	1

n.s.: not significant. All other test statistics are significant, *p* < .0001.

## 5 Results

The grand mean of job transition probability for qualified employees in West Germany during the observed period is about *M* = 12.61% per year, i.e. they change jobs every 8.56 years on the average. This frequency is only slightly higher than for lower qualified white-collar workers in West Germany (8.32 years, *p* < .001) but substantially higher than for qualified employees in East Germany between 1990 and 2010 (9.57 years). Female qualified employees show a different pattern in job transitions than male colleagues. They generally report higher frequencies of job transitions over the years. This holds true for both internal and external transitions. While female internal transition rates have declined from 8.55% in the 1980s to 4.64% nowadays, which is quite similar to male transition rates (3.52%), external transition rates do not show a linear but rather a volatile development over time ranging from 8.57 to 13.25% whereas male rates remain at a stable level between 7.38 and 8.59%. Note that the occurrence of internal job transitions is remarkably low in comparison to external job transitions (Table 1).

The best-fitted models to explain the probability of general job transitions, internal and external transitions are shown in Table 4. We will first present the general model findings (JJ-Trans, left column) before we go more into detail by separating the internal (Int-Trans, middle column) and external transitions (Ext-Trans, right column).

**Table 4:** Best fitted multilevel linear probability models.

Type of effect	JJ-Trans		Int-Trans		Ext-Trans	
	Coeff	SE	Coeff	SE	Coeff	SE
Intercept	.2943⁎⁎⁎	.0158	.1113⁎⁎⁎	.00929	.1736⁎⁎⁎	.01357
*Time related effects*						
Course of time	−.0017⁎	.0008	−.00204⁎⁎⁎	.00047	.00087⁎	.00068
GDP	.0032⁎⁎	.0009	.00056	.00056	.00266⁎⁎	.00080
Lagged GDP	.0037⁎⁎⁎	.0010	.00087	.00059	.00275⁎⁎	.00084
*Individual characteristics*						
Age	−.0086⁎⁎⁎	.0007	−.00290⁎⁎⁎	.00039	−.00516 ⁎⁎⁎	.00057
Sex	.0158 ⁎⁎	.0158	.00835⁎	.00340	.00747	.00513
*Cross-level interaction*						
Time ∗ age	.0001	.0000	.00005 ⁎⁎	.00002	–	–

N = 24 956; non-standardized coefficients.

⁎ *p* < .01.

⁎⁎ *p* < .001.

⁎⁎⁎ *p* < .0001.

The most striking finding is that there is a negative tendency, albeit a slight one, in the frequency of job transitions over time (*b* = − .0017; *p* < .05). Therefore, our Hypothesis 1 postulating no change at all has to be rejected.

Supporting Hypothesis 2, variations in the business cycle have a quite pronounced positive influence on the occurrence of job transitions. The GDP in the year of transition (*b* = .0033; *p* < .001) as well as the lagged GDP of the previous year (*b* = .0035; *p* < .001) provide explanatory value for the probability of job transitions.

Also the age of the qualified employees shows a significant impact on the probability of job transitions. Moreover, the negative age effect as assumed by Hypothesis 3a is by far the strongest effect tested in our model (*b* = − .0086; *p* < .001). Put differently, the probability of a job transition is reduced by 8.6 percentage points every 10 years of one's working life. The assertion in Hypothesis 3b that this age effect is stable over time cannot be generally confirmed. The interaction term of age and the course of time reveal a significant cross-level moderating effect of time and age (*b* = .0001; *p* < .05) meaning a weakened age effect over the years.

Controlling for sex, the data show a higher frequency of job transitions for qualified female employees (1.58 percentage points). This evidence is not moderated by the course of time.

**Fig. 1:**
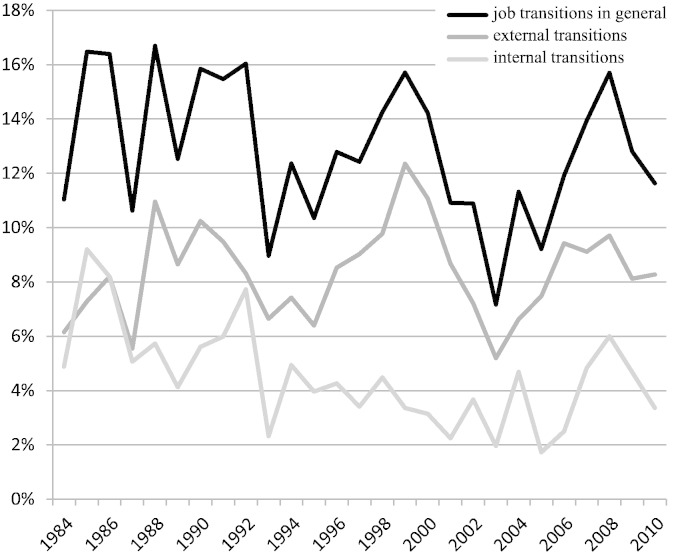
Proportion of job transitions among professional and managerial employees in West Germany from 1984 to 2010.

Fig. 1 breaks the job transitions down into internal and external ones. Besides the volatile pattern of job transition rates over the years, different developments for internal and external transitions are striking. We have conducted identical analyses for both of these subordinated dependent variables. The differentiation uncovers a distinct negative trend over time for internal transitions while the frequency of external transitions has only slightly increased. This main result contradicts the widespread implicit assumption of increasing dynamics in qualified professional and managerial careers, but it confirms a development towards more inter-organizational movements and shrinking internal labor markets. Contrary to the impact of time, the same and the previous year's economic cycles do not affect internal, but only external transitions. Independently of macro-effects, age remains a major regressor. For both internal and external transitions, this negative effect is highly significant, but for external transitions the effect size (*b* = − .0057; *p* < .001) is almost double as high as for internal transitions (*b* = − .0029; *p* < .001). By contrast, the moderator effect of time on age as found in the general model, can clearly be assigned to internal job transitions (*b* = .00005; *p* < .01) whereas the interaction term failed to improve the LPM model for external transitions (Table 3).

## 6 Discussion

At the beginning we posed two questions. (1) Do concepts like the boundaryless career, which have mainly been developed in the Anglo-American context, adequately describe what is happening in different institutional environments such as Germany? (2) To what extent is the underlying change assumption a valid one? The answer to the former is “most probably no”, and to the latter “only to a certain extent”.

As far as the universality of increasing change and job transitions is concerned, our results suggest that the overall level of job transitions among professional and managerial employees in Germany has in fact slightly declined between 1984 and 2011. This is the result of the interplay of internal transitions which have clearly decreased and slightly increasing external transitions. When taken in isolation, both dimensions of physical mobility point in a direction congruent with this mainstream careers theory, but their interplay suggests quite the opposite.

The decrease in internal job transitions indicates shrinking internal, intra-organizational labor markets. These markets used to be one of the central institutions for maintaining employability in systems owing to a logic of vertical coordination (Doeringer & Piore, 1985). Downsizing, delayering and vertical disintegration have truncated the room for internal transitions, especially for highly qualified professionals (cp. Morris & Farrell, 2007). In a number of countries such as Germany (Neus & Walter, 2009) or Australia, New Zealand and South Africa (Littler, Wiesner, & Dunford, 2003) fewer job opportunities are offered by the companies. Delayering also provides an explanation for the perceived cross-level moderator effect of time. Especially middle management with an overrepresentation of younger and middle-aged qualified employees is affected by delayering while elderly professionals are less affected (Littler et al., 2003; see also Faust, Jauch, & Notz, 2000). In addition, employees of big companies are more affected by this development, whereas industry shows a negligible influence over time (Giesecke & Heisig, 2010).

This development is not leveled out by the slight increase in external transitions. Indeed, recent findings from the German language area point towards a revival of, or even a sustained attractiveness of more traditional career aspirations. For example, a longitudinal, multi-cohort study of business school graduates and their career aspirations in Austria indicates that more recent career cohorts show a higher preference for a “company world” career field (Mayrhofer et al., 2011b), characterized by tight coupling and stable configuration (Iellatchitch et al., 2003). In a similar vein, an analysis of career expectations in Germany between 1999 and 2009 shows no tendency towards an increased preference for post-organizational career arrangements (Kattenbach et al., 2011). Arguably, contextual developments like the Russia crisis in 1998 or the so-called financial crisis in 2009 have left their footprint on the career motivations of contemporary individuals. Both aspirations for as well as actual realizations of external transitions are highest in times of economic upturns, since the number of attractive job offers increases and the logic of “chains of vacancy” leads to a leverage effect (Stuck, 2006).

Beyond the lack of increase in the number of job transitions in Germany, the importance of the economic cycle for explaining variations in the level of job transitions supports the need for a contextualized perspective in career studies. We agree with Biemann et al. (2012) that factors like industry size, organizational size, or unemployment rates serve as important variables in the career construction process (see also Savickas, 2005). Another study with a German sample, based on retrospective information on employment histories, found a significant impact of economic growth on inter-organizational mobility as well (Biemann et al., 2011). This fosters the call for comparative career studies with an international or even an inter-continental perspective. Comparing Europe and North America, one should note that the respective business cycles themselves follow different cycle dynamics (e.g. Stock & Watson, 2005). Furthermore the link between the business cycle and labor market may differ in Germany and the US, as labor law in Germany attenuates the business cycle effects, for example by strong protection of employee rights in the case of losing one's job.

As a caveat, an alternative explanation for the general decline in internal changes can be a changed understanding of job transitions themselves. While we have conceptualized an internal job change as any essential change in task or duty, respondents may interpret an internal job change as a clear move onto a different position with a different job title, even if a promotion is not implied. The term ‘job’ might make less sense when working life is regarded as a ‘fluid organization of tasks’ and a constant stream of projects. In this vein, Inkson (1995) noted that the number of professionals who declared a change in duties steadily increased from 2.8% in 1980 to 8% in 1992. Jobs became more flexible and diverse. This could be an explanation for not interpreting job transitions as an internal transition nowadays, because the transition is not regarded as a change in jobs, but rather a change in tasks. In addition, the respective questions in the GSOEP survey may be criticized. Respondents are asked whether they experienced a job change in the last year. Only in the case of confirmation do the respondents have to differentiate between internal change and external change. The questions and their order thus impede the respondents from considering whether changes in their duties are actually a change in their job. Hence, we assume that the low number of internal changes found in the GSOEP may under-represent intra-organizational mobility. This is supported by findings which report higher internal change-rates (e.g. 7.2% between 1987 and 1996 in a case study of a Dutch organization, see Dohmen, Kriechel, & Pfann, 2004; around 9% in a study among professionals in the UK between 1980 and 1992, see Inkson, 1995). Thus the absolute numbers should be judged with caution. Yet, even after taking this caveat into account, there is no reason to suppose that there was a strong increase in the number of overall job transitions during the time span analyzed.

Qualified employees in West Germany change jobs about every 8.5 years on average. Although this number indicates less physical job mobility than in the USA or Japan (Arthur & Rousseau, 1996a), it still illustrates the importance of an inter-organizational career perspective as fostered by the boundaryless career concept instead of focusing on organizational careers. However, as far as the underlying basic change assumption is concerned, we concede that although change takes place, its extent is overestimated. It is a common theme in social science to disagree about the extent to which things change and the volatility “present times” induce (Eccles & Nohria, 1992, p. 25) and to stress that we tend to see “the present … always [as] an exciting, challenging time to be contrasted with a stable past” (Collin, 1998, p. 412). Indeed, Germany's past is in certain aspects different from the present, illustrated e.g. by the increasing number of women entering the labor market (OECD, 2012), or by a greater variety of employment forms and the rise in so-called atypical relations (Brehmer & Seifert, 2008). Yet there are areas of stability as well. For example, results from comparative HRM show that variables like the size of the HR-department relative to the number of employees or the organizational investment in training and development remain stable despite the widespread popular claims to the contrary (Mayrhofer, Brewster, Morley, & Ledolter, 2011). Again, our empirical paper does not conceptually criticize the boundaryless and protean career ideas (to this end, see Arnold and Cohen, 2008, Inkson et al., 2012), but argues for a contextualization thereof (Mayrhofer and Schneidhofer, 2009, Mayrhofer et al., 2007). Such a contextualization encompasses not only countries (Briscoe, Hall, & Mayrhofer, 2012b), but also professions, as e.g. knowledge workers (Colic-Peisker, 2010), academics (Dany, Louvel, & Valette, 2011) or workers in the creative industries (McKinlay & Smith, 2009) experience different developments even in rather regulated contexts.

Future research should, therefore, focus on a more elaborate comparison of the interplay between country, institutional and cultural context, industry, and organizational and individual characteristics to draw a more nuanced picture of developments in career patterns over time. Also, further analyses of the explanatory power of economic growth for different age groups are needed. Preliminary analyses have shown that the GDP has a strong effect on job mobility for young and advanced qualified employees, but not for seniors (Schneidhofer et al., 2012). Moreover, an expansion of the research design to various groups of employees arranged according to e.g. sectors, professions, and work attitudes could provide interesting insights on the scope of our results and reveal additional influential aspects on the development of careers.

## 7 Conclusion

Our results provide support for a critical view on the largely unquestioned acceptance of the assertion that there are increasingly dynamic careers with more frequent job transitions among qualified employees. This does not necessarily challenge the concepts linked with it or the “new deal” at work (Cappelli, 1999). Yet, it cautions researchers neither to generalize too much from observations of specific groups of individuals in specific regions of the world for whom new careers may be a reality, nor to universalize the concept by applying it to diverse cultural and institutional environments. Furthermore, our findings support a call for more comparative research in careers (as realized e.g. in Briscoe, Hall, & Mayrhofer, 2012a) — including qualitative, or even relational (see Özbilgin, 2006) approaches.
